# Understanding stigma: The experiences of people with drug-sensitive pulmonary tuberculosis in Rawalpindi, Pakistan

**DOI:** 10.1371/journal.pone.0324614

**Published:** 2025-06-16

**Authors:** Ruby Stein, Anil Fastenau, Hina Aman, Nimer Ortuño-Gutiérrez, Chris Schmotzer, Sophie CW. Unterkircher, Eva Pilot

**Affiliations:** 1 Department of Health, Ethics & Society, Care and Public Health Research Institute CAPHRI, Faculty of Health, Medicine and Life Sciences, Maastricht University, Maastricht, The Netherlands; 2 German Leprosy and Tuberculosis Relief Association (GLRA/DAHW), HQ, Wuerzburg, Germany; 3 German Leprosy and Tuberculosis Relief Association (GLRA/DAHW), Asian region, India; 4 Aid to Leprosy Patients, Rawalpindi, Pakistan; 5 Damien Foundation, Brussels, Belgium; 6 Department of Global Health, Institute of Public Health and Nursing Research, University of Bremen, Bremen, Germany; Stellenbosch University, SOUTH AFRICA

## Abstract

**Introduction:**

Tuberculosis (TB) is a major global health problem and Pakistan is ranked fifth among the 30 high-burden countries in the world. TB-related stigma affects health seeking behaviour and treatment adherence, increasing disease transmission and worsening health outcomes. This study aimed to explore experiences of stigma among people with TB (PWTB) in Rawalpindi to help inform targeted stigma reduction interventions that could improve health seeking behaviour, treatment adherence and the mental well-being of PWTB in Pakistan.

**Methodology:**

In-depth interviews were conducted with 15 people with pulmonary drug sensitive TB from Rawalpindi, Pakistan. For assessing emerging themes, an inductive themed analysis approach was used. Next, a deductive approach was applied by analysing and interpreting the data against the Health Stigma and Discrimination Framework.

**Results:**

TB- related stigma among participants was driven by fear of infection, which in some cases was due to misconceptions surrounding TB transmission as well as social judgement and gender norms. Stigma manifested through: anticipated and perceived stigma in the form of non-disclosure and fear of social exclusion; enacted stigma among friends and family, in the workplace and healthcare settings; and internalised stigma, The negative outcomes of stigma that resulted for some participants included non- adherence and social exclusion, in the form of loss of marriage prospects and employment.

**Conclusion:**

This study confirms that TB-related stigma persists in Pakistan, impacting he well-being, medication adherence and treatment outcomes of PWTB. The distinct drivers, manifestations and outcomes of stigma in Rawalpindi Pakistan uncovered from this study, supported by previous research, can help inform targeted stigma reduction interventions such as public education programmes.

## Introduction

Tuberculosis (TB) is a major global health problem and one of the leading causes of death worldwide [1]. TB is preventable and most people who develop TB can be cured with timely diagnoses and the correct treatment. Despite this, an estimated 10.6 million people fell ill with TB worldwide and a total of 1.6 million people died from TB in 2021 [2]. Pakistan is ranked fifth among the 30 high-burden countries in the world [3]. In 2021, the total TB incidence rate in Pakistan was estimated to be 611,000 and 50,100 deaths were estimated to be attributed to TB. There were a total of 343 024 PWTB notified in 2021 [2]. The treatment coverage of the total PWTB was estimated to be 55%. The treatment success rate of newly diagnosed and relapsed PWTB registered in 2020 was 94% [2]. The state of Punjab, where Rawalpindi is located, has the highest number of PWTB in Pakistan, with 96,619 out of 167,787 people with pulmonary TB notified in 2019) [4]. Globally and in Pakistan, TB-related stigma has been identified as a significant factor impacting health seeking behaviour and treatment adherence [5–8] as well as the mental well-being of PWTB [9,10], which can lead to increased disease transmission, Multi-Drug Resistant TB (MDR-TB) and mortality [11–13].

Stigma is very context specific and therefore, stigma research to inform targeted stigma reduction interventions needs to consider location specifications [14]. Although quantitative research has underlined the alarming impact of TB stigma in Pakistan, this study aims to address the dearth of research which explores the in-depth experiences of stigma among PWTB through a qualitative approach. This study uses the “The Health Stigma and Discrimination Framework” [1] to explore the drivers and facilitators, stigma manifestations and outcomes among people with drug-sensitive pulmonary TB in Rawalpindi, Pakistan. The use of this framework aims to help inform tailored stigma reduction interventions that have the potential to improve health seeking behaviour; treatment adherence; treatment outcomes and the mental well-being of PWTB.

## Methodology

### Study design

A qualitative study design involving semi-structured open ended interviews was applied to this research. The definition of stigma used in this study was informed by Goffman [15] who defines stigma as “*an attribute that is deeply discrediting*”. He describes a stigmatized person as being “*tainted*” and “*discounted*’ in the eyes of society. Current conceptualizations often classify health related stigma into four categories. 1. *Perceived stigma* refers to the belief that those without the condition perceive people with the condition negatively; 2. *Anticipated stigma* refers to expectations of discrimination, stereotyping, and/or prejudice from others post disclosure due to a stigmatized condition; 3. *Enacted stigma* refers to actual experiences of discrimination, stereotyping, and/or prejudice from others in the past or present due to a stigmatizing condition; and 4. *Internalized stigma* refers to individuals endorsing negative feelings and beliefs associated with the stigmatized condition and applying them to themselves [8,16–18]. Differentiating between stigma mechanisms (enacted, anticipated, perceived and internalised stigma) has the potential to help inform targeted interventions [19]. The construction used for this study is “The Health Stigma and Discrimination Framework” [1] which underlines the different dimensions of stigma and their manifestation for TB patients and health impacts.

### Study setting

The fieldwork was conducted in Rawalpindi Leprosy Hospital in the district of Rawalpindi, Pakistan. Rawalpindi is located in the north of the Punjab Province and next to the capital of Pakistan; Islamabad. There are 14 treatment centres in the Rawalpindi District which treated slightly above 10,000 PWTB in 2022. In 2022, there was a total of 531 DS- TB patients registered in Rawalpindi Leprosy Hospital. 519 of those cases began treatment in Rawalpindi Leprosy Hospital. The treatment success rate was 82%. 34 patients died and 22 cases were lost to follow up. 91 patients were not evaluated or transferred out of the hospital. In Pakistan, there are no designated mental health services for DS-TB patients. Each Programmatic management of DR-TB (PMDT) site has a psychologist who has to assess the patient at the beginning of treatment and during treatment. Rawalpindi Leprosy Hospital has an understanding with the department of Psychiatry at a Government Teaching Hospital to ensure adequate psychiatric services for people with DR-TB in need (information obtained through email conversations with Rawalpindi Leprosy Hospital, March 2023).

### Study population and participant selection

The study population consisted of pulmonary people with DS- TB visiting Rawalpindi Leprosy Hospital for treatment. A purposeful sampling method was applied by the researcher to include people with pulmonary TB, DS-TB and those on TB treatment for at least four months. Other criteria included those who were above 18 years old and those willing to participate. A greater interest among females to participate resulted in a gender imbalance (SI Table 1).

### Data collection

Recruitment of participants started on the 4^th^ of July until the 19^th^ of September 2022. 15 in-depth interviews were conducted among PWTB at Rawalpindi Leprosy Hospital. All interviews took place in person in Urdu.

An interview guide was developed for the interviews and informed by evidence from literature and stigma assessment toolkits [20–22]. The interviews took place in a separate quiet room to ensure adequate privacy and to provide a space where the participants felt comfortable to give honest feedback. The questions in the interview guide (SI Table 2) were designed in a way to capture the different dimensions of stigma (perceived, anticipated, enacted, and internalized) experienced by participants [8]. The interviewers (HA) professional background and training as a psychologist allowed for active listening, empathy, and interpreting verbal and nonverbal cues, which enabled her to ask insightful questions and probe deeper into responses..

The interviewer used subtle probes to encourage areas of thought. The interview lengths varied between 10–40 minutes.

After the interviews took place, they were translated from Urdu to English. Accordingly, as new insights emerged, modifications were made to the topic guide. All interviews were audio-taped with a voice recorder on a smartphone.

### Ethical considerations

Informed consent process was applied to all participants, during which they were told that participation was entirely voluntary, the information obtained would be strictly confidential and it would not impact their medical care. Participants were also warned about the potential risks of participation, including recounting painful memories. The interviewers background as a psychologist also allowed her to manage the sensitive topics and situations of emotional distress, ensuring the well-being of participants.

Participants were informed that if needed they can take a break at any given time or stop the interview completely. Psycho-social support is available to DR-TB patients at Rawalpindi Leprosy Hospital and these services were made available and underlined to the study participants if necessary. Written consent was obtained from all participants. For those who could not read or write, the consent form was read out loud in the presence of a witness, and a thumb impression taken in lieu of a signature. Only researchers involved in the direct study and the professional translator, who was asked to sign a non-disclosure agreement, were allowed to access the collected data. All interviews that took place were recorded and transcribed anonymously to ensure confidentiality. In Pakistan, the institution of the principle investigator must provide ethical clearance for a research study. Accordingly, prior to data collection, ethical approval was obtained through the Global Health Master’s Ethical Review Committee from Maastricht University, registration number FHML/GH_2022.041.

### Data analysis

The transcriptions were used as a basis for the thematic analysis. Firstly, an inductive approach was used by attaching codes to the entire data set and looking out for emerging themes [23]. The qualitative research analysis software called ATLAS.ti was used to facilitate data analysis. Next, a deductive approach was applied by analysing and interpreting the data against “The Health and Discrimination Framework” [1]. The identified codes were then merged into themes and fitted within the framework.

## Results

Interviews took place with 15 people with drug-sensitive TB who were attending Rawalpindi Leprosy Hospital for treatment (SI Table 1). One participant was accompanied by her sister, who also facilitated answering the interview questions. The demographics of this participant were not recorded.. The findings are supported by selected quotations where participant IDs are referred to. TB patients discussed their experiences of stigma and incidents that friends, family members or other PWTB shared with them during the course of their illness or hospital visits or stays. Participants had significantly varying experiences of stigma. For example, some patients reported no experiences of stigma and only positive support from friends and family members.

### Theme one: Drivers and facilitators

#### Fear of illness and infection.

Fear of infection, often due to misconceptions about TB transmission, was a significant driver of stigma among participants. Participants discussed their own fear associated with illness and death due to TB (Int 5, 8,10, 11) and passing the illness onto loved ones (Int 2, 10, 11, 13) as well as others fear of infection (Int 2, 11). One participant states:

*“I was afraid while thinking if I would be able to survive or not. I was also concerned that it will be transmitted to other family members as there were children also in my family”* (Woman 45, int 10)

In some cases, fear of infection seemed to be driven by misconceptions around TB transmission. Participants discussed some of their own misconceptions and those of people around them, including the idea that there is no cure for TB or that TB will reoccur once you get it once (Int 8, 14), that it can be passed through sharing utensils (Int 2,6, 7, 15), cooking for someone (Int 15) or through touching the clothing of a person with TB (Int 2, 7).

Participants also spoke about their experiences of loved ones not fearing illness or carrying out precautionary measures due to religious beliefs (Int 2, 5). For example, one participant shares:

*“My children and husband didn’t distance themselves from me. My children said that what happens to a person is from Allah Almighty”* (Woman 48, int 5)

#### Social judgement and gender norms.

Social judgement and gender norms can result in some of the manifestations of stigma discussed in the section below. Participants highlight that tuberculosis “*is not taken in a positive sense in our (Pakistani) society”* (Int 4) and that *“people relate a disease to the persons sins”* (Int 2). One participant expressed “*feeling afraid (upon diagnosis) while thinking about societies perception (of them)”* (Int 13).

Participants gave examples of direct experiences of blame for their TB diagnosis from friends: “*Some of my friends would taunt me saying that I should have refrained from wrong activities. They were of the view that I suffered from TB because of my own actions”* (male 22, int 6) and in a local doctor’s office, after informing him about her diagnosis, he said: “*Oh! I don’t know why people get infected with this disease. I think this is nothing but the result of their sins”* (Woman 28, Int 13).

Gender norms seemed to play a significant role in the distinct experiences of stigma among participants. Woman discussed feeling pressure to be (and appear) healthy (Int 4) and to have the ability to carry out household tasks such as cleaning the house, washing the dishes and cooking the food (Int 4, 7, 11, 13 15). One participant shares her experience of being a woman with TB:

***“****My husband was of the view that a woman should be healthy and of fair complexion. I was healthy and had fair complexion, but my colour became dull because of TB. As a result, he wouldn’t take much care of me (…) I realised that I must be going through the changes which are causing him to divert his attention (…) Obviously, you will have to face the music by your in-laws and husband if you are unable to take care of your house (…) Most of the time, women have to face these problems (…) An affected woman is unable to give time to her husband and children properly.”* (Woman 26, Int 4)

Participants also discussed the distinct challenges that men face because of the pressure on them to be earning a livelihood for the family (Int 6, 13, 15). For example, one participant shares their belief that men face greater challenges due to the increased likelihood of people being aware of their diagnosis from having responsibilities outside the home:

*“Men are outside the house and women are inside the house, even if they (women) talk, they will talk only at home. As you know, a man has to see many people outside the house. If one comes to know about the disease, he will tell the others. I live in a village.”* (Woman 22, Int 6)

### Theme two: Manifestations of stigma

#### Anticipated and perceived stigma.

Participants discussed expectations of stigma that resulted from the drivers explored above, leading to non-disclosure and fear of social exclusion. Many participants reported keeping their diagnosis from people due to the fear of being stigmatised (Int 1, 2, 9, 10, 11, 12, 13, 14, 15). Some participants only informed their close family members of their diagnosis, such as their partners, parents and siblings.

#### Enacted stigma.

Participants provided examples of experiences of stigma from family members, friends, colleagues and health professionals. It is important to note that the majority of participants highlighted positive support from close family members e.g., through consolation after hearing about their diagnosis, encouragement and speaking positively about the possibility of good health outcomes, visiting or accompanying the participant in the hospital, calling to enquire about their well-being, caring for them and helping them eat and drink when unwell, as well as some also providing examples of support from friends and extended relatives, other patients, employers and medical staff. One participant states:

*“It will help others to know how a person with TB should be dealt with. A person with TB shouldn’t be abandoned. Otherwise, they will die. Praise be to Allah; my husband and daughters took care of me.”* (Woman 42, Int 2)

Though one participant described abandonment from her husband and his family, stating that her husband’s family advised him to divorce her after learning about her TB diagnosis:

*“They all abandoned me after I suffered from this disease. No one is there to support me”*(Woman 61, Int 12)

Some also described experiencing stigma in the form of neglect, abonnement and excessive precautions from wider family members and neighbours (Int 2, 11, 12, 13). For example, from the words of two participants:

*“I had good relations with my neighbours, but they also distanced themselves after coming to know about my disease. Everyone started hating me and stopped visiting me”* (Woman 42, Int 2)

*“From their faces, I could read that they weren’t happy to see me at their homes and seemed as if they were telling me to visit them after recovering from TB. Upon my visit, many of the relatives would restrict themselves into their rooms to give me an indication that I shouldn’t visit them next. They even wouldn’t ask me if I wanted to drink water, take tea or have meal. Perhaps, they weren’t willing to share their utensils with me”* (Women 28, Int 13)

Enacted stigma can also take place in the workplace. One participant shared an experience of their cousin who also had TB:

*“In the case that he would forget to use his own bowl at his workplace, the people there would tell him to go out and throw that bowl and buy a new one for them. He shared that these things made him worried at first and he started thinking to quit the job” (*Women 28, Int 13)

Enacted stigma in health facilities was also highlighted among participants through forced social distance (Int 1, 13), blame (Int 13) and rude behaviour (Int 4): One participant described their experience in a pharmacy:

*“They (pharmacy staff) told me to stay away, and also said that this disease (TB) was more lethal than coronavirus.” (Male, Int 1)*

### Internalised stigma

People also often internalised society’s negative beliefs and the fear and stigma associated with TB. One participant reported blaming themselves as a reaction to stigmatisation from society:

*” I thought that perhaps I didn’t adopt precautionary measures properly and that’s why I was suffering from it once again.”* (Woman 42, Int 11)

Another participant described that they became severely depressed during the first month of her illness. She was fearful that her parent- in-laws would convince her husband to take her son away from her care because she had heard stories about women being abandoned by their family after getting ill:

*“I used to be in depression. Even, I wasn’t able to dress my son properly*” (Woman 28, Int 13)”

### Theme three: Outcomes

#### Non-adherence.

One participant in this study opened up about their experience not adhering to medication one month after diagnosis:

*“I realized that they (neighbours) would think I was still suffering from TB if I continued visiting the hospital for my medication, but they will blame me for making lame excuses if I tell them I just have an infection and nothing more than that (…) Once, I decided not to take the medication anymore. So, I visited the hospital and returned to my home without taking medicine. I want to quit medication so as to make them think that I just had a minor infection now and no more TB, but my husband came to know about what I had done”* (Woman 28, Int 13)”

She began adhering to medication again after a few days due to her husband’s support:

*“I let him know that I am okay and that it was useless to take any medicine (…) He said that I shouldn’t pay attention to what people say and said my health condition may worsen if I quit medication all of sudden”* (Woman 28, Int 13)”

### Societal exclusion (loss of marriage prospects and employment)

Another major outcome of TB stigma is exclusion from society. Participants reported incidences (and fear) of missed social events, loss of marriage prospects and job loss which cam have detrimental impacts on the mental well-being of PWTB and impact their livelihood.

One participant described an incident of a called off marriage proposal due to her TB diagnosis and highlighted the effect this can have on the wider family, leading her to thoughts of self-harm:

*“It leaves a negative impact when the elder sisters is rejected. Everyone in the wider family/society comes to know that the elder sisters was rejected because of TB. So people avoid (the person with TB) while declaring that family as the one infected with TB (…)I felt like harming myself, but I avoided it because of my sisters. I didn’t want to leave a negative impact on them”* (Woman 21, Int 14)

Four participants in this study discussed how they lost their job or were replaced after TB diagnosis (Int 2,8, 9, 12). Three participants discussed how they received positive support from their employers and continued to receive their salaries from their place of work when they were diagnosed with TB and could not work due to weakness or ill health (Int 1, 6, 14).

## Discussion

To address the process prior to the application of stigma, interventions should aim to target the drivers and facilitators of stigma [1]. The results highlight a need for general TB education programmes for the community in Pakistan to ensure awareness about the curable nature of TB, reduce social judgement and blame and tackle misconceptions to limit stigma associated with unrealistic fears [24–26]. While some precautions carried out towards PWTB can be considered appropriate, many of the precautions that were carried out by loved ones, colleagues and health professionals were excessive and illegitimate. For example, isolating or distancing from individuals beyond what is considered the standard time for people with DS-TB to be infectious (2–3 weeks after starting treatment depending on health), or carrying out health precautions in excess of standard precautions e.g. avoiding sharing utensils or touching the clothing of a person with TB [2,24].

Research suggests that enacted stigma in healthcare settings is particularly detrimental for individuals, with it increasing anticipated stigma, impacting treatment adherence and affecting engagement with care [27,28]. This means that PWTB who have experienced stigma from healthcare professionals in the past may be less likely to access healthcare in the future due to the fear and expectation of being stigmatised. Regular refresher courses in TB control and management for health professionals should be implemented to address the fear of infection [27]. Health workers should also be mindful to provide the correct information to patients. For example, they should not tell TB patients to use separate utensils as this can lead to rejection or isolation and stigma and it is not necessary [24,29,30]. Future research should aim to understand health professionals knowledge of methods of TB transmission and explore the advice that they are providing to PWTB.

This study showed that societal judgement and gender norms can also drive the manifestations and outcomes of stigma experienced by participants. Similar to other studies in different settings [8,28–30], the results illustrated that women in Pakistan can be viewed as “undesirable” when appearing unwell with TB and/ or if they are unable to carry out the household tasks socially expected from them as wives, mothers or daughters. This can result in diminished marriage prospects for young women and their family members and in some cases lead to divorce, which was the experience for one women in this study. In Pakistan, women are often financially dependent on their husbands or male family members. Therefore, income-generation programs for women living with TB, and the promotion of education and employment among women, has the potential to extend women’s roles beyond the domestic sphere and reduce some of the distinct TB associated issues that women face [29].

This study supports the array of research that suggests that TB stigma negatively impacts treatment compliance [8,28,31]. As seen in the results, non-adherence can occur directly from the fear of being stigmatised. However, depression and poor mental wellbeing in itself is also widely known as a factor contributing to non-adherence [9,32]. Prior research suggests PWTB who have higher (internalised and perceived) stigma are more likely to develop depression [9,10]. This study supports a need for mental health services for DS-TB patients, alongside DR-TB patients. Research suggest that the integration of mental health services, including peer-led support groups, into existing TB facilities can improve symptoms of depression and anxiety, and increase treatment completion rates [33–35]. Common obstacles to the provision of adequate mental health services for PWTB should be addressed, such as inadequate prioritisation, limited research, low levels of awareness and lack of training [36]. Research exploring insights from implementers can help to understand specific obstacles to the provision of mental health services for people with DS TB in Pakistan

### Limitations

There are a number of limitations of this study. The experiences of PWTB who did not seek treatment or adhere to medication for less than four months were not included in this study. Future research should include the perspectives of PWTB who have not adhered to treatment to further explore reasons for non-adherence and to better understand stigmas role in this.. This study took place in a hospital setting with a current staff member. Therefore, patients may have been hesitant to share any experiences of stigma within the hospital setting. The data was not representative of both genders, with most participants being female. This sample interviewed TB patients exclusively. It would have been beneficial to include the perspectives of health professionals and family members. Furthermore, this study took place during the COVID- 19 pandemic, which potentially compounded or altered participant’s stigma experiences. Future research should also explore the relationship between COVID-19 and TB and further examine the impact of COVID-19 on TB-related stigma.

## Conclusion

The results of this study support that TB-related stigma can impact the well-being, medication adherence and treatment outcomes of PWTB, as well as contribute to disease transmission e.g. through non-disclosure. There is a clear need for stigma reduction interventions, such as: educational interventions; mental health services for DS-TB patients, alongside DR-TB patients; seeking help from local religious leaders to dispel myths associated with TB and peer support for patients to improve treatment outcomes and mental health. Future research should explore the perspectives of health professionals, PWTB who have not adhered to treatment or been lost to follow up, people with DR-TB and extra-pulmonary TB and the policy perspective from implementors e.g.to explore implementation challenges. Furthermore, gender specific differences in TB-related stigma in Pakistan should also be examined further.

## Supporting information

SI Table 1 Participants Demographic Information. (DOCX)

SI Table 2 Interview Guide. (DOCX)
