# Supplementary material for: Understanding stigma: The experiences of people with drug-sensitive pulmonary tuberculosis in Rawalpindi, Pakistan
Source: PLoS One. 2025 Jun 16;20(6):e0324614. doi: 10.1371/journal.pone.0324614 (PMC12169514; doi:10.1371/journal.pone.0324614)
Supplement: SI Table 1 — (DOCX) [file pone.0324614.s001.docx]

SI 1 Table 1: Participants demographic information

| **Sex** |  | **Marital Status** |  | **Religious Affiliation** |  | **Family Structure** |  | |
| --- | --- | --- | --- | --- | --- | --- | --- | --- |
| Female | 11 | Married | 12 | Muslim | 8 | Nuclear | 6 | |
| Male | 4 | Single | 3 | Christian | 1 | Joint | 5 | |
|  |  |  |  | Unknown | 6 | Unknown | 4 | |
|  |  |  |  |  |  | Children | yes | no |
|  |  |  |  |  |  |  | 10 | 5 |
|  |  |  |  |  |  |  |  | |
| **Employment status** |  | **Education level/ literacy level** |  | **Yearly income** |  | **Age group** |  | |
| Housewife | 7 | Illiterate | 3 | 10k-20k | 2 | 18-24 years | 3 | |
| Employed | 4 | Middle school (grade 1-8) | 4 | 20k-30k | 6 | 25-34 years | 3 | |
| Unemployed due to TB | 2 | Matriculation (grade 9-10) | 3 | 30k-40k | 1 | 35-44 years | 4 | |
| Unknown | 2 | Intermediate (grade 11-12) | 4 | 50k+ | 1 | 45-54 years | 3 | |
|  |  |  |  | Unknown | 4 | 55-64 years | 1 | |
|  |  |  |  |  |  | 65 and above | - | |
|  |  |  |  |  |  | Unknown | 1 | |
