# Supplementary material for: Understanding stigma: The experiences of people with drug-sensitive pulmonary tuberculosis in Rawalpindi, Pakistan
Source: PLoS One. 2025 Jun 16;20(6):e0324614. doi: 10.1371/journal.pone.0324614 (PMC12169514; doi:10.1371/journal.pone.0324614)
Supplement: SI Table 2 — (DOCX) [file pone.0324614.s002.docx]

| **Theme**  **SI 2 Table 2: Interview Guide** | **Interview question** |
| --- | --- |
| **Demographics**  **(can be filled out by interviewer from prior knowledge or through asking participants)** | Age:  Sex:  Education:  Occupation:  Monthly Income:  Medical status:  Family structure (joint family system or nuclear family):  Marriage status:  Number of children: |
| **Introduction of the interviewer** | **Read out information form and obtain consent here (see information form and consent form below)**  “The main goal of this interview is to see things the way you see them…it's more like a conversation with a focus on your experience, your opinions and what you think or feel about the topics covered in the questions I ask” |
| **Ice breaker** | **Start out with less sensitive questions to break the ice**  For example:  How are you doing?  Where are you coming from today?  What brings you here today? |
| **Transition** | Thanks so much for sharing that. Now that we have gotten to know each other a little better, I’d like to move into some of the main topics that we want to cover in this interview. |
| **Knowledge, attitudes and practices about TB + TB diagnostic journey** | Can you tell me about your illness (TB)?  How did you feel when you heard you were diagnosed with TB? Can you explain why you felt this way?  Before you were diagnosed, what were your thoughts about TB?  Can you tell me about your treatment seeking process from the time you got diagnosed until now?  Probe: When did you first engage with TB care after being diagnosed? |
| **Disclosure** | Have you told your family / friends / neighbours that you have TB?  Were you afraid to tell anyone that you have TB?  Probe: Why were you afraid? (e.g. due to societal stigma)  Can you tell me about their reaction? How did you feel after you told them? Did you expect this reaction? |
| **Transition** | Some of the aspects about having TB that you have already mentioned (or that many people talk about) are stigma (the negative ways that that people are viewed or feel because of their differences) and discrimination (being treated badly by others because of these differences). I would like to talk more about these topics now. I understand that these are sensitive topics, and some of these questions may bring to mind painful experiences. You are welcome to share only what you feel comfortable with, and can stop or take breaks at any time. |
| Experience in treatment and care | Can you tell me about your experience taking TB medication and visiting Rawalpindi Leprosy Hospital and other clinics since your TB diagnosis?   - Probe: how is your relationship with providers, what do you think about the quality of care you receive?   What barriers have you faced when trying to access care or take your medicine ? (e.g. commuting barriers) how have you dealt with these barriers?   - Probe for positive and negative experiences, always ask for examples   What other medical support have you received (e.g., counselling, grant support, food or transport assistance, community health worker, social work, etc.)?   - Probe: how do you experience these supports? Do you find them helpful?   Do you have any preferences or suggestions on further enhancing TB services going forward? Or If you could change something what would it be? |
| **Consequences of TB stigma and coping mechanisms** | How has having TB impacted your relationships with family and friends? How has this affected you?  How has having TB impacted your access to income? How has this affected you?  What do you do to comfort yourself from the negative impact that TB can have on your mental well-being ? (e.g. talking to family, listening to music, watching TV)  Who do you talk to for comfort or support? |
| **Perceptions and attitudes about TB** | How has your perception about TB changed over time (since being diagnosed, starting treatment)?  What do you think others (your community) think or feel about TB patients?  What stereotypes do you hear about people affected by TB?  Do you believe there is a difference with how men and women who have TB experience stigma? |
